# The Therapeutic Effect of Traditional LiuJunZi Decoction on Ovalbumin-Induced Asthma in Balb/C Mice

**DOI:** 10.1155/2021/6406295

**Published:** 2021-09-29

**Authors:** Wenting Xu, Rui Zhao, Bin Yuan

**Affiliations:** ^1^The First Clinical Medical College, Nanjing University of Chinese Medicine, Nanjing 210046, Jiangsu Province, China; ^2^Department of Traditional Chinese Medicine, Hainan Women and Children's Medical Center, Haikou 570206, Hainan Province, China; ^3^Department of Pediatrics, Affiliated Hospital of Nanjing University of Chinese Medicine, Nanjing 210029, Jiangsu Province, China

## Abstract

**Aim:**

To investigate the therapeutic effect of LiuJunZi decoction (LJZD) in an experimental model of asthma and uncover its potential mechanism.

**Materials and Methods:**

The ovalbumin (OVA) was applied to induce asthma in Balb/C mice, and LJZD was orally administrated to asthmatic mice. The lung function and histological lesion were evaluated by airway hyperresponsiveness assay, lung edema assay, and hematoxylin and eosin staining. The amounts of CD4^+^CD25^+^Foxp3^+^ T_Reg_ cells were analyzed through combining fluorescent antibody staining with flow cytometry assay. The levels of inflammatory factors and immunoglobulins were detected by enzyme-linked immuno sorbent assay (ELISA). The expression of miR-21 and miR-146a was investigated by real-time PCR. The protein expression of activating protein-1 (AP-1), nuclear factor kappa-B (NF-*κ*B), and NF-*κ*B inhibitor alpha (I*κ*B*α*) was determined by western blotting.

**Results:**

LJZD improves OVA-induced asthma in Balb/C mice, which is manifested by decreasing lung edema, Penh levels, lung histological lesion, and inflammatory cell infiltration. LJZD increased the number of CD4^+^CD25^+^Foxp3^+^ T_Reg_ cells in blood mononuclear cells from asthmatic mice. Furthermore, LJZD reduced the levels of tumor necrosis factor-*α* (TNF-*α*), interleukin- (IL-) 4, IL-6, IgG1, and IgE, but increased interferon gamma (IFN-*γ*) expression, in serum of asthmatic mice, and also decreased the expression of IL-17a, IL-23, IL-25, and thymic stromal lymphopoietin (Tslp) in lung tissues. In addition, miR-21 and miR-146a expression and phospho (p)-NF-*κ*B, p-I*κ*B*α*, and AP-1 protein expression were inhibited by LJZD in lung tissues from asthmatic mice.

**Conclusion:**

LJZD improved OVA-induced asthma in Balb/C mice by inhibiting allergic inflammation and Th2 immunoreaction, which might be associated with the inactivation of the NF-*κ*B signaling pathway.

## 1. Introduction

Asthma, characterized by the chronic inflammation, constriction, and high reactivity of the airway, has been affecting 300 million people worldwide and will have affected almost 400 million people by 2025 [1–4]. Although the exact cause of asthma has not been identified, the genetic, immunologic, and environmental risk factors mostly account for this epidemic disease [5–7]. Most asthmatics suffer type 2 inflammation regulated by T-helper 2 (Th2) lymphocytes, and the Th2 lymphocytes induce the secretion of lgE antibody and inflammatory cytokines, during the development of asthma [8, 9]. These inflammatory cytokines subsequently induce the production of mucous glands, collagen deposition, and smooth muscle dysfunction in the airway, which leads to airway remodeling. The serious airway remodeling will result in the loss of lung function which will produce great economic and life burden of asthmatics. Although *β*-agonist, bronchodilators, and corticosteroids temporarily relieve the asthmatic symptoms, the inefficiency to control asthma progression and various cardiovascular, bone, and gastrointestinal side effects of the abovementioned medicines makes their use limited [10, 11]. Therefore, developing safe and efficient therapeutic medicines for asthma has always been a vital concern and challenge for researchers.

Traditional Chinese medicine (TCM) with long-term clinical application exerts effectiveness for improving asthma with no obvious side effects [12]. In TCM, multiple ingredients work on comprehensive body targets to synergistically develop the pharmaceutical effects in the treatment of asthma [13]. LiuJunZi decoction (LJZD), composed of Pilose Asiabell Root, Largehead Atractylodes Rhizome, Indian Buead, Liquorice Root, Tangerine Peel, and Pinellia Tuber, is a classic TCM compound for invigorating the spleen and revolving qi [14]. The pharmacological research proved that LJZD can ameliorate chemotherapy-induced side effects, functional dyspepsia, and chronic atrophic gastritis [15–17]. Besides, LJZD and its ingredients also protect lung function from smoke-induced chronic obstructive pulmonary disease and improve the allergic response through regulating the physiologic function of the DC-CD4^+^ T-cell interaction in the dust mite allergy asthma patients [18–20]. Therefore, we aim to comprehensively evaluate the therapeutic effect of LJZD on asthma and identify its potential mechanism, which may provide us some enlightenment on the treatment of asthma.

In this study, the lung function and histological lesion were evaluated in an ovalbumin- (OVA-) induced asthma model of Balb/C mice. The levels of inflammatory factors and immunoglobulins and the expression of miR-21 and miR-146a in serum and lung tissues from asthmatic mice were detected. The protein expression of activating protein-1 (AP-1), nuclear factor kappa-B (NF-*κ*B), and NF-*κ*B inhibitor alpha (I*κ*B*α*) in lung tissues was also determined. The results indicated that LJZD improves OVA-induced asthma by inhibiting allergic inflammation and Th2 immunoreaction, and the mechanism might be related to the inactivation of the NF-*κ*B signaling pathway.

## 2. Materials and Methods

### 2.1. Reagents

OVA was purchased from Alfa Aesar Chemical Co., Ltd. (Karlsruhe, Germany). Dexamethasone (DXM), Al(OH)_3_, phorbol 12-myristate 13-acetate (PMA), and ionomycin were all obtained from Sigma-Aldrich Co., Ltd. (St. Louis., MO, USA). Allophycocyanin-conjugated anti-mouse CD4 antibody (APC-CD4), fluorescein isothiocyanate-conjugated anti-mouse IL-17 antibody (FITC-IL-17), phycoerythrin-conjugated anti-mouse CD25 antibody (PE-CD25), and the corresponding isotype controls were bought from eBioscience Inc. (San Diego, CA, USA). Brefeldin A was purchased from BD Pharmingen (San Diego, CA, USA). Enzyme-linked immuno sorbent assay (ELISA) kits for mouse interleukin- (IL-) 4, IL-6, IL-17a, IL-23, IL-25, tumor necrosis factor-*α* (TNF-*α*), and interferon gamma (IFN-*γ*) were bought from Bio-Swamp Life Science Lab (Wuhan, China). ELISA kits for total-lgE and lgG1 were obtained from BOSTER biological technology Co., Ltd. (Wuhan, China). Antibodies against AP-1 and actin beta (Actb) and HRP-conjugated goat anti-mouse secondary antibody were provided by Bioss Inc. (Beijing, China). Antibodies against phospho (p)-NF-*κ*Bp65 (Ser536), NF-*κ*Bp65, p-I*κ*B*α* (Ser32), and I*κ*B*α* were purchased from Cell Signaling Technology (Denver, MA, USA). All reagents for cell culture were acquired from Invitrogen Co., Ltd. (Paisley, UK). Other chemical reagents were of analytical grade.

### 2.2. Ethics

All animal procedures were performed according to the guidelines for the use and care of American Laboratory Animals (NIH Publication No. 85-23, revised in 1985). Also, animal experiment was approved by the Committee on the Ethics of Animal Experiments of Nanjing University of Chinese Medicine.

### 2.3. Preparation of LJZD

Six herbs including Pilose Asiabell Root, Largehead Atractylodes Rhizome, Indian Buead, Liquorice Root, Tangerine Peel, and Pinellia Tuber were bought from Tong Ren Tang Pharmaceutical Co., Ltd., (Beijing, China) and met the Pharmacopoeia standard (version 2015). The composing proportion of Pilose Asiabell Root, Largehead Atractylodes Rhizome, Indian Buead, Liquorice Root, Tangerine Peel, and Pinellia Tuber was 9 : 9: 9 : 6: 3 : 4.5 by weight. Every 20 g materials were sliced and then placed in 300 mL water. The LJZD was made by the water boiling method, until the drug solution was 200 mL. The LJZD was cooled to room temperature for usage, and the administration dosage to mice was calculated by converting the adult dosage according to the body surface area conversion factor.

### 2.4. Animals

48 female Balb/C mice (five weeks old, 20–25 g) were bought from Huafukang Bio-Technology Co., Ltd. (Beijing, China). All mice were adapted to the housing environment (40–60% humidity, 24 ± 2°C, and 12-hour light/dark cycles) for one week before experiments. All mice were free to eat basal mouse chow and drink water.

### 2.5. Establishment of the OVA-Induced Asthma Model

The Balb/C mice were randomly separated into 4 groups (12 mice per group): the control (Con), model (Mod), DXM, and LJZD treatment groups. The mice in the Mod, DXM, and LJZD groups were intraperitoneally injected 0.2 mL 10% OVA/Al(OH)_3_ mixed solution at the 0th, 7th, and 14th day for sensitization. After that, 1% OVA saline was administrated as nasal drops to the Mod, DXM, and LJZD to induce asthmatic mice from the 21st to 27st day. For the Con group, the saline was applied for sensitization and stimulation. Then, the saline was intragastrically administrated to Con and Mod mice, while 0.7 mg/kg DXM and 12, 6, 3 mL/kg LJZD were orally provided to DXM and high-, medium-, and low-dosage LJZD groups from the 28st to 34st day, respectively. The DXM group was applied as the positive control. The mice were anesthetized with intraperitoneal injection of sodium pentobarbital (35 mg/kg) and then sacrificed by cervical dislocation on the 35st day.

### 2.6. Airway Hyperresponsiveness (AHR) Assay

All mice were stimulated by inhaling 0, 10, 20, 30, 40, and 50 mg/mL aerosolized methacholine for 3 min, respectively. Then, the mice were placed in a closed system to observe the enhanced pause (Penh), and the Penh was applied to calculate the AHR.

### 2.7. Lung Edema Assay

After sacrifice, the right lungs were dissected and weighted as wet weight (*W*). Then, the lungs were subjected to oven drying at 56°C for 15 min, and the dry weight (*D*) was recorded. The *W*/*D* ratio was applied to evaluate the lung edema.

### 2.8. Pathological Histology

The lungs were immersed by 4% paraformaldehyde, followed by dehydration, paraffin embedded, and sectioned with 3 *µ*m thickness with an Ultra-Thin Semiautomatic Microtome (Leica, Bensheim, Germany). After that, they were deparaffinized with xylene solution and stained with hematoxylin and eosin (H & E) according to the standard procedures. The histological images were captured by using a digital camera (Leica microsystems Inc., Buffalo Grove, IL, USA).

### 2.9. Blood Mononuclear Cell (BMC) Collection and Cell Culture

The peripheral blood samples were collected from the orbit and centrifuged at 500 rpm for 5 min. The precipitation was applied to prepare peripheral blood BMCs by the Ficoll-Hypaque density gradient centrifugation method. The BMCs were cultured in RPMI 1640 medium supplemented with 100 U/mL penicillin, 0.1 mg/mL streptomycin, and 10% (V/V) foetal bovine serum (FBS) in a 37°C humidified incubator with 5% CO_2_. 5 × 10^5^ BMCs were stimulated by 1 mmol/L ionomycin, 1 mg/mL PMA, and 10 *µ*g/mL brefeldin A for 4 h before flow cytometry analysis.

### 2.10. Flow Cytometry

The stimulated BMCs were washed by phosphate buffered saline (PBS) three times, and then, 1 × 10^5^ cells were incubated with 2 *μ*L of APC-CD4 and 2 *μ*L of PE-CD25 at 4°C for 20 min in a dark place. Then, the cells were thoroughly washed by PBS and permeabilized with 0.1% Triton X-100 for 5 min. The cells were performed intracellular staining for forkhead box P3 (Foxp3) through being incubated with 1 *μ*L of FITC-IL-17. The staining procedures were conducted in accordance with the manufacturer's instruction. The stained cells were detected by flow cytometer (Beckman Coulter, Brea, CA, USA) and analyzed by FlowJo software (Tree Star, Ashland, OR, USA).

### 2.11. ELISA

Serum was acquired by centrifugation at 4°C, 2000 rpm for 10 min. The serum cytokines including IL-4, IL-6, TNF-*α*, and IFN-*γ* and immune proteins lgE and lgG1 were detected by using ELISA kits, according to the instructions of the manufacturer.

1 mg of lung tissues was homogenized in 5 mL ice-cold PBS through a glass homogenizer. The tissue homogenates were centrifuged at 5000 rpm, 4°C for 10 min, and supernatants were applied for IL-17a, IL-23, IL-25, and thymic stromal lymphopoietin (Tslp) detection by using ELISA kits, according to the instructions of the manufacturer.

### 2.12. Real-Time PCR

Total RNA in lung tissues was extracted by Trizol reagent (Invitrogen; Carlsbad, CA, USA), according to the manufacturer's instructions. 1 *μ*g of tissue RNA was reversed transcribed to synthesize cDNA by using a SuperScript III RT kit (Invitrogen), and real-time PCR (RT-PCR) was performed by using the SYBR^MT^ qPCR Mix (Invitrogen). Specific primers used for genes are shown in Table 1. The Actb and U6 small nuclear 1 (Rnu6) were applied as the internal control for mRNA and miRNA, respectively, and the relative gene expression intensity was calculated by the 2^−∆∆Ct^ method.

### 2.13. Western Blotting

The protein samples were extracted by lysing 1 mg of lung tissues in 1 mL of ice-cold RIPA buffer for 30 min and centrifugated at 20,000 rpm, 4°C for 15 min. The protein extractions were boiled at 100°C for 5 min before western blotting. 20 *µ*g protein of each sample was subjected to 12% SDS-PAGE and then transferred to PVDF membranes. The membranes were blocked by 10% goat serum and then incubated with primary antibodies diluted in TBST: AP-1 (1 : 2000), *β*-actin (1 : 2000), p-NF-*κ*Bp65 (Ser536) (1 : 1000), NF-*κ*Bp65 (1 : 1000), p-I*κ*B*α* (Ser32) (1 : 1000), and I*κ*B*α* (1 : 1000), overnight at 4°C. The next day, PVDF membranes were incubated with HRP-conjugated secondary antibodies at room temperature for 2 h, and then, proteins were visualised by Immobilon Western Chemiluminescent HRP Substrate detection reagent (Millipore, Bedford, MA, USA).

### 2.14. Statistical Analysis

Statistical analyses were conducted by using Statistical Product and Service Solutions 27.0 software (SPSS Inc., IL, USA) and GraphPad Prism 5.0 (GraphPad, CA, USA). Data were presented as the mean ± standard deviation (SD), and two-tailed, unpaired Student's *t*-test was used to compare data between two groups. To compare data from multiple groups, one-way analysis of variance with the Bonferroni post test was applied. The *p* < 0.05 was considered statistically significant.

## 3. Results

### 3.1. LJZD Improved OVA-Induced Asthma in Balb/C Mice

**Figure 1:**
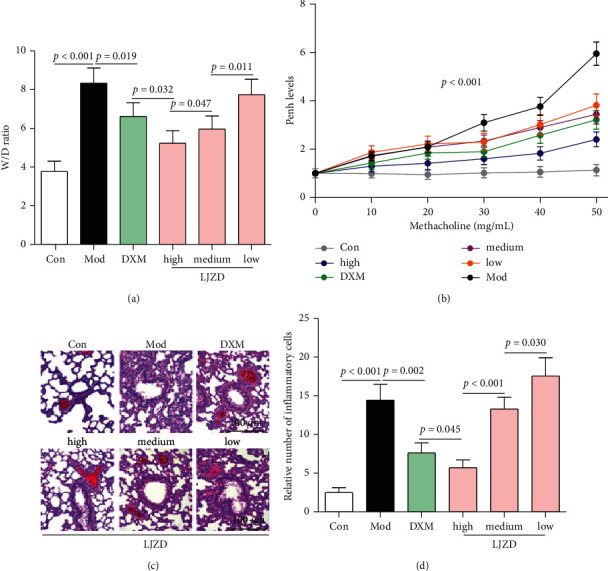
LJZD improved OVA-induced asthma in Balb/C mice. (a) The *W*/*D* ratio of the right lungs in the Con, Mod, DXM, and high-, medium-, and low-dosage LJZD groups. (b) The Penh levels represented air hyperresponsiveness of mice in all the groups. (c) Representative H & E staining images of the right lungs in all the groups. (d) The relative number of inflammatory cells in H & E staining of all the groups. Data were expressed as the mean ± SD (*n* = 3). LJZD: LiuJunZi decoction, OVA: ovalbumin, *W*/*D*: wet weight/dry weight, Con: control, Mod: model, DXM: dexamethasone, H & E: hematoxylin and eosin, and SD: standard deviation.

Compared with the Con group, OVA stimulation induced higher W/*D* ratio in the Mod group, which suggested severe lung edema in asthmatic mice (Figure 1(a), *p* < 0.05). Compared with the Mod group, LJZD effectively decreased the *W*/*D* ratio in a concentration-dependent manner (*p* < 0.05), which indicated that LJZD treatment could inhibit OVA-induced lung edema. Furthermore, the Penh levels were increased in the Mod group compared with the Con group, while LJZD treatment could decrease Penh levels in a concentration-dependent manner (Figure 1(b), *p* < 0.001). From the H & E staining, the inflammatory cell infiltration, thickened airway epithelium, and mucous membranes were clearly observed in the Mod group (Figure 1(c)). Simultaneously, LJZD treatment effectively ameliorated inflammatory cell infiltration in a concentration-dependent manner (Figure 1(d), *p* < 0.05).

### 3.2. LJZD Increased the Number of CD4^+^CD25^+^Foxp3^+^ Cells in BMCs of Asthmatic Mice

**Figure 2:**
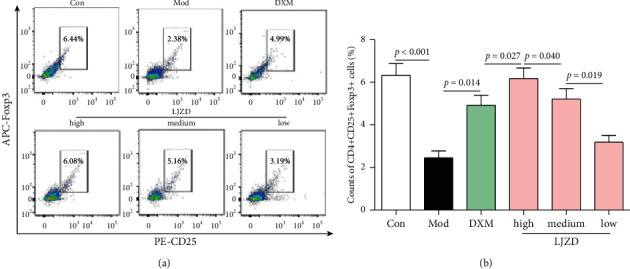
The representative images (a) and amounts (b) of CD4^+^CD25^+^Foxp3^+^ cells in the Con, Mod, DXM, and high-, medium-, and low-dosage LJZD groups by combining fluorescent antibody staining with flow cytometry assay.

The amounts of CD4^+^CD25^+^Foxp3^+^ regulatory-suppressor T cells (CD4^+^CD25^+^Foxp3^+^ T_Reg_ cells) in BMCs were decreased in the Mod group compared with those of the Con group (Figure 2, *p* < 0.05). Furthermore, LJZD treatment effectively increased the quantities of CD4^+^CD25^+^Foxp3^+^ T_Reg_ cells in BMCs in a dose-dependent manner (*p* < 0.05).

### 3.3. LJZD Regulated Inflammatory Factor, lgG1, and lgE Secretion in Asthmatic Mice

**Figure 3:**
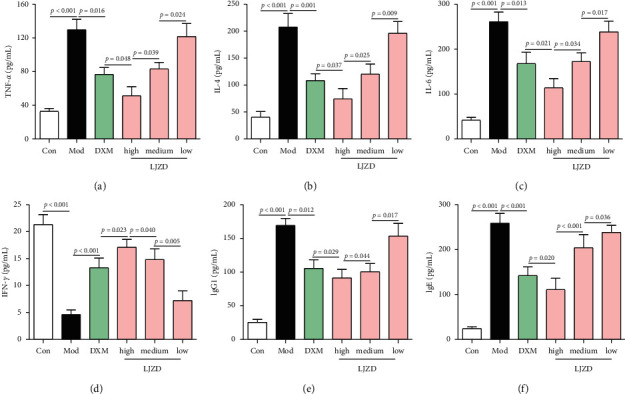
LJZD regulated TNF-*α*, IL-4, IL-6, IFN-*γ*, lgG1, and lgE secretion in asthmatic mice. The protein levels of TNF-*α* (a), IL-4 (b), IL-6 (c), and IFN-*γ* (d) in and serum of the Con, Mod, DXM, and high-, medium-, and low-dosage LJZD groups were detected by ELISA. The levels of lgG1 (e) and lgE (f) in serum of all the groups were also detected by ELISA. The results were shown as the mean ± SD from at least three independent experiments. TNF-*α*: tumor necrosis factor-*α*, IL: interleukin, IFN-*γ*: interferon gamma, and ELISA: enzyme-linked immuno sorbent assay.

Results of ELISA showed that the levels of TNF-*α*, IL-4, and IL-6 were increased, but IFN-*γ* level was decreased in the serum of the Mod group compared with that of the Con group (Figures 3(a)–3(d), *p* < 0.05). Interestingly, the levels of TNF-*α*, IL-4, and IL-6 were decreased, but IFN-*γ* level was increased by LJZD in a dose-dependent manner (*p* < 0.05). In order to investigate the effect of LJZD on Th2 inflammation, the Th2-related immunoglobulins, lgG1 and lgE, were determined. The secretion of lgG1 and lgE was raised in the serum of the Mod group compared with that of the Con group, while LJZD effectively decreased the levels of lgG1 and lgE in a dose-dependent manner (Figures 3(e) and 3(f), *p* < 0.05).

**Figure 4:**
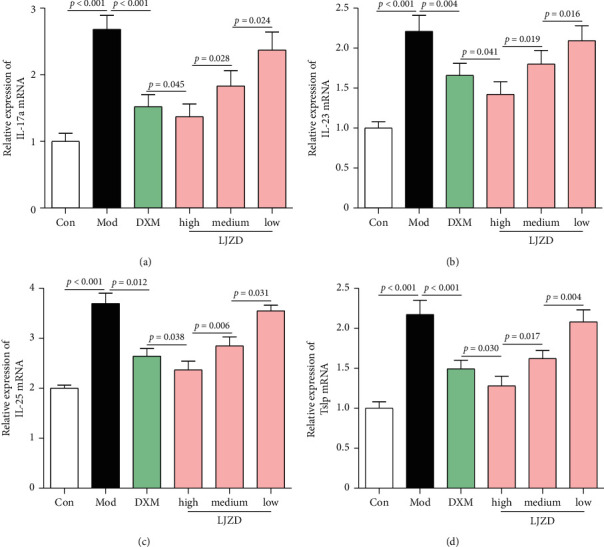
LJZD reduced the mRNA expression levels of IL-17a (a), IL-23 (b), IL-25 (c), and Tslp (d) in lung tissues of asthmatic mice by real-time PCR. Data were expressed as the mean ± SD (*n* = 3). Tslp: thymic stromal lymphopoietin.

**Figure 5:**
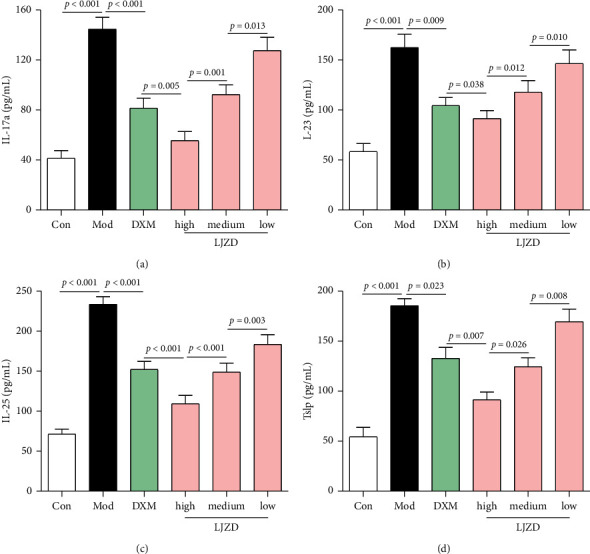
LJZD decreased the protein expression levels of IL-17a (a), IL-23 (b), IL-25 (c), and Tslp (d) in lung tissues of asthmatic mice by ELISA. Each value represented the mean ± SD from at least three independent experiments.

In addition, results of real-time PCR revealed that higher expression levels of IL-17a, IL-23, IL-25, and Tslp were found in the lung tissues of the Mod group compared to the Con group (Figures 4(a)–4(d), *p* < 0.05). The mRNA expression of IL-17a, IL-23, IL-25, and Tslp was decreased by LJZD administration in a dose-dependent manner (*p* < 0.05). Similarly, ELISA assay showed that the protein concentrations of IL-17a, IL-23, IL-25, and Tslp in lung tissues were inhibited by LJZD in a dose-dependent manner and high-dosage LJZD exerted better effect than DXM (Figures 5(a)–5(d), *p* < 0.05).

### 3.4. LJZD Reduced miR-21 and miR-146a Expression in Asthmatic Mice

**Figure 6:**
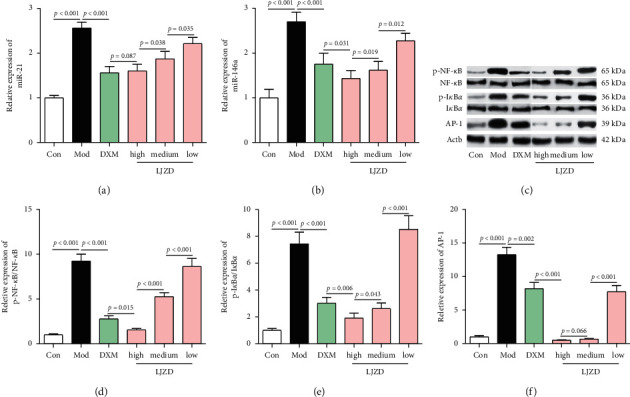
LJZD reduced miR-21 and miR-146a expression and suppressed the activation of the NF-*κ*B signaling pathway. Real-time PCR analysis of the miR-21 (a) and miR-146a (b) expression in lung tissues of the Con, Mod, DXM, and high-, medium-, and low-dosage LJZD groups. (c) The representative images of western blotting analysis of NF-*κ*B, phospho (p)-NF-*κ*B, I*κ*B*α*, p-I*κ*B*α*, and AP-1 expression in lung tissues of all the groups. The relative expression levels of p-NF-*κ*B (d), p-I*κ*B*α* (e), and AP-1 (f) are shown, and Actb was used as an internal control. Data were expressed as the mean ± SD (*n* = 3). NF-*κ*B: nuclear factor kappa-B, I*κ*B*α*: NF-*κ*B inhibitor alpha, and AP-1: activating protein-1.

The expression of miR-21 and miR-146a was increased in lung tissues of the Mod group compared with that of the Con group (Figures 6(a) and 6(b), *p* < 0.05). LJZD inhibited the expression of miR-21 and miR-146a in a dose-dependent manner (*p* < 0.05).

### 3.5. LJZD Suppressed AP-1 and the Phosphorylation of NF-*κ*B and I*κ*Ba Protein Expression in Asthmatic Mice

From the western blotting, the expression of AP-1 was upregulated and the phosphorylation of NF-*κ*B and I*κ*Ba expression was increased in the Mod group compared with that in the Con group (Figures 6(c)–6(f), *p* < 0.05). Moreover, LJZD exerted the dose-dependent suppression on AP-1 and phosphorylation of NF-*κ*B and I*κ*Ba expression (*p* < 0.05).

## 4. Discussion

During the development of allergic inflammation, T_Reg_ cells exert immunosuppressive activity and regulate inflammatory cytokine profiles, in addition to Th1 and Th2 cells [21]. Thymus-selected CD4^+^CD25^+^Foxp3^+^ T_Reg_ cells are a subset of T_Reg_ cells which can suppress the allergic type 2 inflammation, and the decrease of these cells indicated suppressed immunologic response in peripheral blood and destructed peripheral tolerance to allergens [22]. Therefore, CD4^+^CD25^+^Foxp3^+^ T_Reg_ cells are considered as one kind of target cells for allergen-specific immunotherapy in asthma [23]. The inflammatory cytokines TNF-*α*, IL-4, and IL-6 in blood can attack tissues and organs. Besides, IL-4 and IL-6 secreted by Th2 cells directly induce the generation of asthma-specific lgG1 and lgE, thus intensifying Th2 differentiation and regulating Th1/Th2 dynamic balance [24]. In this study, we found that LJZD effectively regulated the amounts of CD4^+^CD25^+^Foxp3^+^ T_Reg_ cells, inflammatory cytokines, and immunoglobulins, which suggested that LJZD could improve the peripheral immunologic process, increase peripheral tolerance to allergens, and inhibit Th2 inflammation in the development of asthma.

The infiltrated epithelial and inflammatory cells secreting inflammatory cytokines in the airway are greatly initiated by IL-17a, while IL-23 can induce the secretion of IL-17a [25]. Not only IL-17a but also many abovementioned inflammatory factors can activate NF-*κ*B and I*κ*Ba and promote the expression of AP-1. NF-*κ*B, I*κ*Ba, and AP-1, as important transcription factors, are responsible for modulating inflammation and immune response in various cells [26]. Therefore, the high expression of IL-17a and IL-23 deteriorates airway inflammation and accelerates inflammatory cell infiltration. Th2-lymphocyte factor-induced inflammation disease can be regulated by IL-25 and Tslp which promote the initiation of Th2 inflammation in airway mucosa, through facilitating Th2-cell proliferation and differentiation as well as activating group 2 innate lymphoid cells [27–29]. Besides the Th2 inflammation modulating effect, IL-25 and Tslp also play vital roles on the airway remodeling through acting on airway smooth muscle cells [30]. In our study, LJZD suppressed the expression of these asthmatic alarming cytokines, which indicated that LJZD could improve the Th2 inflammation and lung tissue remodeling.

Although the influence of various microRNAs in the progress of asthma is still controversial, many research studies validated that microRNAs, such as miR-21, miR-155, and miR-146a, played important roles in the regulation of airway epithelium and smooth muscle function and immunomodulation [31]. miR-21 is related to the maintenance of the immune homeostasis. Lu et al. [32] found that miR-21 level is increased in allergic airway inflammation and regulates IL-12p35 expression. Recently, research supported that miR-21 deficiency decreases eosinophil levels in mice blood [33]. Similarly, our results found the upregulation of miR-21 in lung tissues of asthmatic mice. Shi et al. [34] reported that miR-146a participates in the asthma process, mainly by regulating the inflammatory cytokines. Notably, miR-146a expression was increased in asthmatic mice, and this finding is contrary to a previous study which suggested that miR-146a expression is downregulated in allergic rhinitis mice [35]. The reason might be related to histological difference. Furthermore, our results revealed that the expression of miR-21 and miR-146a was inhibited by LJZD treatment, which confirmed that miR-21 and miR-146a expression are associated with asthma.

Increasing literature has convincingly demonstrated a clear role for NF-*κ*B transcription factors in asthma [36]. AP-1 is one of the main transcription factors that are involved in the regulation of procollagen-*α*1I promoter via inhibiting its activity. Jacques et al. [37] reported that the overexpression of AP-1 impairs corticosteroid inhibition of collagen production by fibroblasts isolated from asthmatic subjects. Recent evidence has suggested that NF-*κ*B and AP-1 are considered as promising targets for pharmacological approaches in asthma therapy [38]. Interestingly, our results found that LJZD treatment decreased the protein expression of AP-1 and the phosphorylation of NF-*κ*B and I*κ*B*α* in lung tissues from asthmatic mice. These data demonstrated LJZD improved OVA-induced asthma in Balb/C mice, and the mechanism might be related to the inactivation of the NF-*κ*B signaling pathway.

This study has some limitations. The relationship between NF-*κ*B and inflammatory factors is unclear due to no blocking up of the NF-*κ*B signaling pathway. The mechanism of the improvement effect of LJZD for asthma may be associated with other signaling pathways. Although high-dosage LJZD exerted better therapeutic effect for asthma than DXM, these data were only confirmed in a mouse model, and whether it is really effective for the treatment of asthmatic patients in clinic remains unknown. Therefore, follow-up research will focus on solving these problems.

In conclusion, LJZD improved OVA-induced asthma in Balb/C mice by inhibiting allergic inflammation and Th2 immunoreaction, which might be associated with the inactivation of the NF-*κ*B signaling pathway. LJZD might be a promising regimen for asthmatic therapy in the future.

## Figures and Tables

**Table 1:** The primer sequences for real-time PCR.

Genes	Forward (5'-3')	Reverse (5'-3')
IL-17a	ACTACCTCAACCGTTCCA	CTGCCTCTGAATCCACATT
IL-23	AATAATGTGCCCCGTATCCAGT	GCTCCCCTTTGAAGATGTCAG
IL-25	AGAGGGCCAGGTGTACAATC	TGCCACAACAGCATCCTCTA
Tslp	ACATTTGCCCGGAGAACAAG	TGCCATTTCCTGAGTACCGT
Actb	GTGACGTTGACATCCGTAAAGA	GCCGGACTCATCGTACTCC
miR-21	TAGCTTATCAGACTGATGTTG	CAGTGCAGGGTCCGAGGTA
miR-146a	CCTGTGAAATTCAGTTC	CAGTGCAGGGTCCGAGGTA
Rnu6	CTCGCTTCGGCAGCACA	AACGCTTCACGAATTTGCGT

IL: interleukin, Tslp: thymic stromal lymphopoietin, Actb: actin beta, Rnu6: U6 small nuclear 1.

## Data Availability

The data used to support the findings of this study are available from the corresponding author upon request.
